# An Extremely Rare Case of Birk-Barel Syndrome With Severe Central Apneas

**DOI:** 10.7759/cureus.15862

**Published:** 2021-06-23

**Authors:** Michael A Ramirez-Arenalde, Wilmarie J Bruckman-Blanco, Abymael Frontanes-Heredia, Sherry L Santiago-Castro, Wilfredo De Jesús-Rojas

**Affiliations:** 1 Internal Medicine, Ponce Health Sciences University, Ponce, PRI; 2 Pediatrics, Centro Médico Menonita de Cayey, Cayey, PRI; 3 Pediatrics, Universidad Central del Caribe School of Medicine, Bayamón, PRI; 4 Pediatrics, Ponce Health Sciences University, Ponce, PRI; 5 Pediatrics, University of Puerto Rico, Medical Sciences Campus, San Juan, PRI; 6 Pediatrics, San Juan Bautista School of Medicine, Caguas, PRI

**Keywords:** birk-barel syndrome, kcnk9, task3, imprinting, central apneas, hypotonia, non invasive ventilation, intellectual disability, puerto rico, maternal inheritance

## Abstract

Birk-Barel syndrome, alternatively known as KCNK9 imprinting syndrome, is caused by a missense mutation in the potassium two pore domain channel subfamily K member 9 (KCNK9) gene on chromosome 8q24.3. This syndrome demonstrates dominant inheritance and is imprinted with paternal silencing, where the paternally inherited allele is silenced, and the maternally inherited allele is active. Congenital hypotonia, palatal abnormalities, intellectual disability, severe feeding difficulties, and dysmorphic facial features characterize this sporadic genetic syndrome. To date, there are approximately 21 molecularly diagnosed individuals worldwide described in the literature. We describe the first known case of Puerto Rican ethnicity, a 16-month-old female born prematurely at 36-weeks with Birk-Barel syndrome, confirmed with whole-exome sequencing, and her response to non-invasive ventilation as a treatment for her sleep breathing disorder.

## Introduction

Birk-Barel syndrome, also known as KCNK9 imprinting syndrome, was first described in 2008 by Barel et al. [1]. This syndrome demonstrates autosomal dominant inheritance with paternal imprinting. It is caused by a specific missense mutation 770G>A in exon 2, replacing glycine at position 236 by arginine (G236R) in the maternal copy of KCNK9 within this locus [2]. KCNK9 encodes a two pore-domain leak potassium channel TASK3, which regulates the resting membrane potential and influences action potential duration and neuron firing frequency [3]. These channels are found in higher concentrations in neurons, especially neurons of the cerebellum. TASK3 channels modulate the excitability of cells and play an essential role during the development of the central nervous system [3].

Clinical presentation is characterized by congenital central hypotonia, severe feeding difficulties, delayed development and intellectual disability, and dysmorphic facial manifestations. Diagnosis is established in a proband with suggestive clinical findings and the detection of heterozygous KCNK9 pathogenic variant p.Gly236Arg on the maternal allele by molecular genetic testing [4]. To date, approximately 21 patients have been molecularly diagnosed and reported in the literature, with 15 of them belonging to the original Arab-Israeli family reported by Barel et al. [1]. We describe the first case of Birk-Barel syndrome of Puerto Rican ethnicity and her response to non-invasive ventilation as a treatment for her central apneas. This case report was previously presented as a poster at the 39th Annual Research and Education forum MSC, University of Puerto Rico, Medical Science Campus on April 10-12, 2019.

## Case presentation

We present a unique case of a 16-month-old female brought to a community hospital in Puerto Rico due to cough and difficulty breathing which developed one day before admission. She subsequently developed acute respiratory failure with hypoxemia and was admitted to the pediatric intensive care unit (PICU). According to her history, the patient is the first child of healthy non-consanguineous Puerto Rican parents and was born prematurely at 36 weeks of gestation by vaginal delivery after an otherwise uncomplicated pregnancy. Birthweight was 1.87kg (3rd percentile), and height was 40.6cm (<3rd percentile). Upon birth, the patient was noted to have congenital hypotonia, cleft palate, abundant oral secretions, bilateral hand contractures, clitoromegaly and talipes equinovarus feet. After birth, she was admitted to the neonatal intensive care unit (NICU) for 28 days due to respiratory distress and poor oral coordination. There was no family history of any genetic disorders or congenital anomalies.

**Figure 1:**
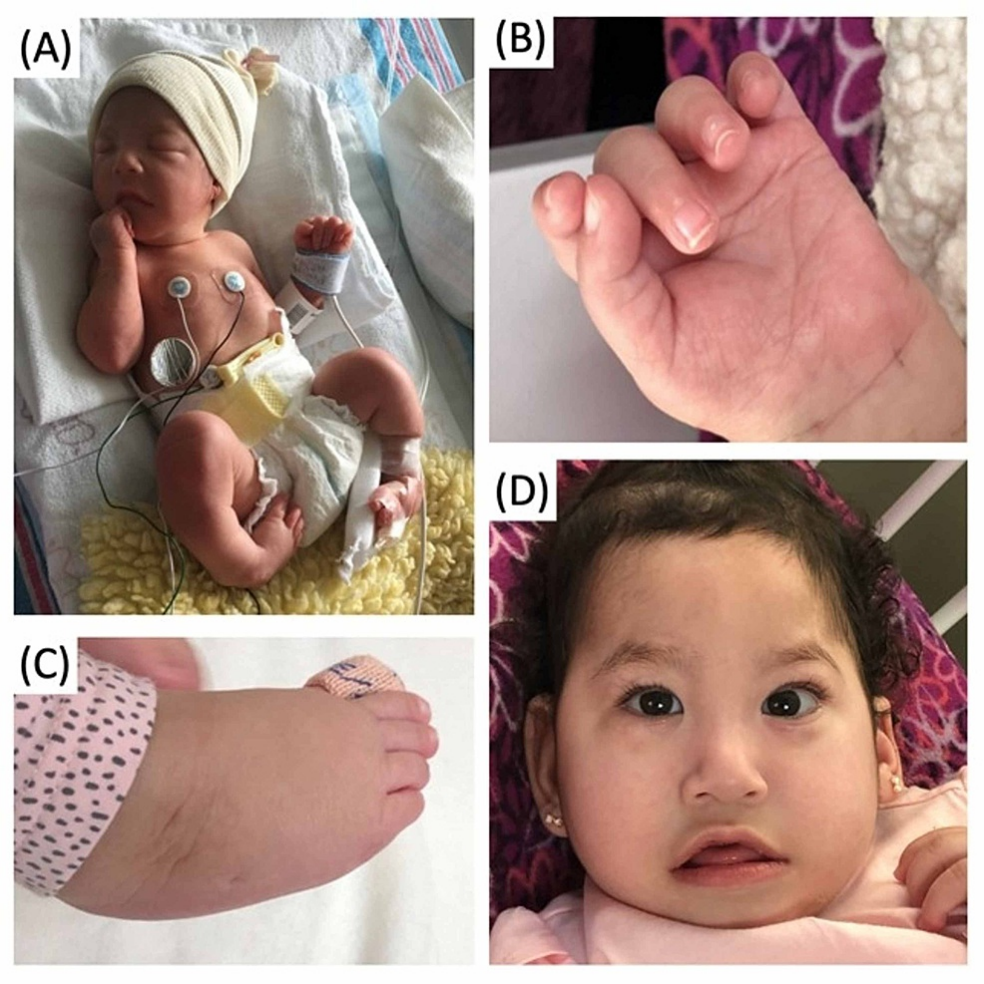
Physical Examination Characteristics of Birk-Barel Syndrome (A) Premature baby on day 1 of life. Bilateral feet deformities consistent with congenital talipes equinovarus. (B) Tapered fingers.  Single palmar crease present. (C) Talipes Equinovarus: inclined inwards, axially rotated outwards, and pointing downwards foot. (D) Note bitemporal narrowing, elongated face, short philtrum, outward-pointing upper lip (tented lip), broad base short philtrum, retromicrognathia, long eyelashes, epicanthal folds and thin neck. Cleft palate was also present. Figure reproduced with permission from Dr. De Jesús-Rojas.

Past medical history included: failure to thrive, congenital talipes equinovarus, global developmental delay, gastro-oesophagal-reflux disease and two previous hospitalizations due to bronchiolitis. In addition, the patient had a gastrostomy for long-term nutrition due to hypotonia, dysphagia and poor oral coordination. Physical examination was also relevant for generalized hypotonia, left medial eye strabismus, cleft palate, mild to moderate pectus excavatum, weak cry, tapered fingers, single palmar crease, and bilateral clubfeet. In addition, Dysmorphic facies were noted: bitemporal narrowing, elongated face, broad-based short philtrum, retromicrognathia, long eyelashes and epicanthal folds (Figure 1). Due to patient symptomatology, medical history, and abnormal clinical picture, further examination was performed.

**Figure 2:**
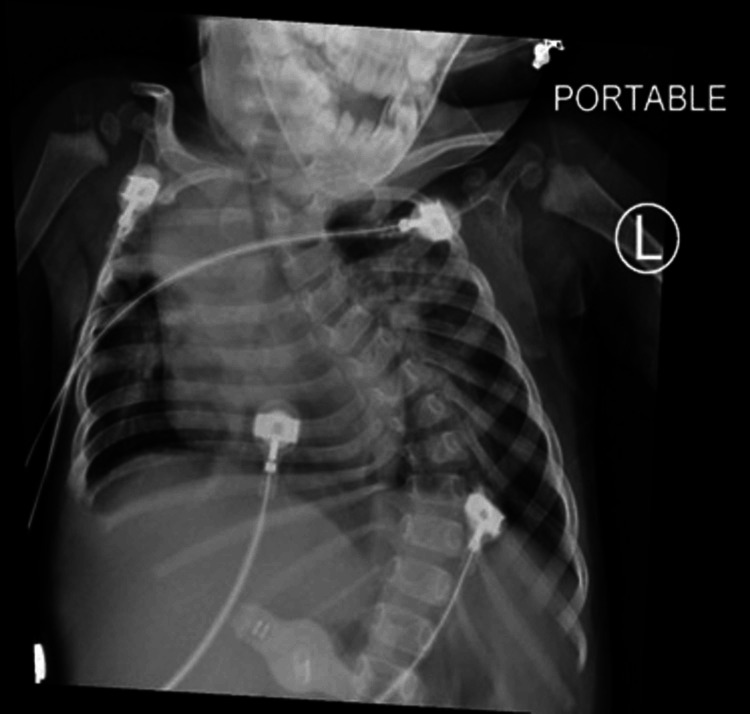
Chest X-ray, portable of a patient with Birk-Barrel Syndrome Bilateral perihilar opacities with right upper lobe atelectasis. Abnormal curvature of spine consistent with severe dextroscoliosis.

**Figure 3:**
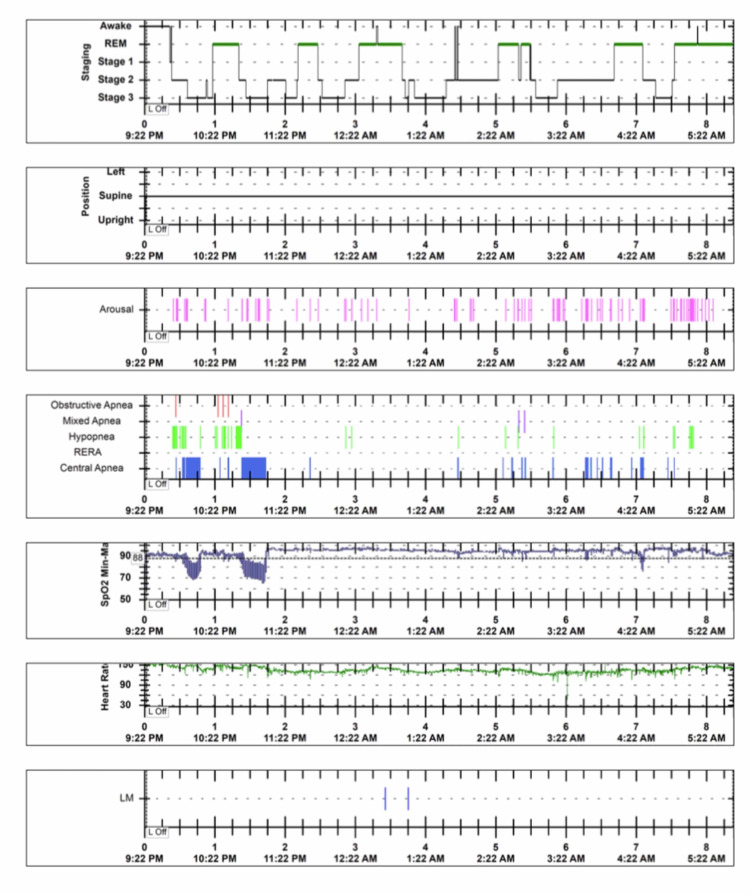
Hypnogram Initial diagnostic polysomnography of our case with Birk-Barel syndrome. Polysomnography demonstrated 4 obstructive-apneas, 3 mixed-apneas and 214 central-apneas with an apnea-hypopnea index (AHI) of 35.9 events per hour. Figure reproduced with permission from Dr. De Jesús-Rojas

Radiographic imaging showed perihilar infiltrates, severe dextroscoliosis and mild adenoidal hypertrophy (Figure 2). Polysomnography showed severe central sleep apnea with an apnea-hypopnea index (AHI) of 35.9 events per hour (Figure 3). Video electroencephalography monitoring and basic laboratories and metabolic workup were found normal. After the initial sleep study, the patient was given supplemental oxygen at night with a 1 litre per minute (LPM) nasal cannula. In PICU, considering severe apneas and low levels of arousal, non-invasive ventilation (NIV) with nasal mask interface with Bi-level positive pressure were given while asleep. Supplemental oxygen was provided to keep saturations above 92%. Additionally, a regimen with albuterol, hypertonic saline 3.5% and chest physiotherapy every 8 hours was provided as part of her airway clearance regimen. After 15 days on NIV with an oxygen flow rate of 1 LPM, progressive improvement in her motor and social milestones were observed, with improved levels of alertness and interaction with caretakers and increased smiling frequency. Whole-exome genetic testing subsequently resulted in a heterozygous mutation in the KCNK9 gene on chromosome 8q24.3, which confirmed the clinical diagnosis of KCNK9 imprinting syndrome. Subsequently, the patient was discharged with established diagnosis and recommendations to involve a multi-disciplinary team: follow up with physical medicine and rehabilitation, speech therapist, Neurology, Gastroenterology, Pulmonologist and Ophthalmology for ongoing clinical management aims of improvement of quality of life. A follow up diagnostic polysomnography at 3-years-old showed interval worsening of both obstructive and central apneas. A total of 22 obstructive sleep apneas, 52 mixed apneas, 199 obstructive hypopneas and 416 central apneas were recorded. Patient AHI was 117.3 events per hour. The minimum oxygen value was 73% due to secondary central apneas. Findings were consistent with hypoxemia due to severe obstructive and central apneas. 

## Discussion

Birk-Barel syndrome is a sporadic genetic disorder, with approximately 21 cases described in the literature. Etiology is thought to be due to maternally inherited pathogenic variants associated with the KCNK9 imprinted gene, which leads to the same amino-acid exchange p.Gly236Arg [2]. This specific amino acid exchange reduces the outward current of the TASK3 channel by approximately 80%[4]. A total of 19 of 21 published cases demonstrate this mutation. However, a novel variant c.710 > A: p.Ala237Asp in the KCNK9 gene and mosaicism 46,XY,r(8)(p23q24.3)/45, XY,−8 have been reported in a 17-year-old girl and a 2.5-year-old boy, respectively [5,6]. This underlines the importance of continued proband studies and genetic testing to elucidate possible alternative mutations or mechanisms of disease development.

With the pathogenic variant p.Gly236Arg, our patient demonstrated the characteristic clinical presentation of congenital hypotonia, dysmorphic facies, cleft palate, intellectual disability, and feeding problems that warranted gastrostomy placement and sleep disturbances due to both central and obstructive sleep apneas [2]. Additionally, our patient exhibited left eye medial strabismus and bilateral equinovarus feet; these features have not been described in previous Birk-Barel syndrome patients but have been observed in patients with ring chromosome eight [7, 8]. Additional clinical characteristics associated with KCNK9 imprinting syndrome but not observed in our patient include clonus, seizures, thin neck, pilonidal dimple or sinus, filar cyst, and transient neonatal hypoglycemia with hyperinsulinism, and decreased lacrimation [2].

To date, no specific management guidelines have been developed, and management remains primarily supportive. However, due to multi-systemic clinical manifestations, a multi-disciplinary team of specialists should be involved for treatment, surveillance, and continuity of care. After confirmed diagnosis through genetic testing, there are some surveillance recommendations for patient care. These recommendations include assessment of nutrition evaluation for nutritional status and growth every six months until age two years, then annually; ophthalmology assessment for decreased lacrimation at least annually; Otorhinolaryngology and gastroenterology evaluation for feeding difficulties every six months until age two years, then annually. Nasogastric or gastrostomy tube placement should be considered if severe dysphagia is present to minimize probabilities of recurrent aspiration [4]. Yearly sleep studies are recommended with follow up with sleep medicine to monitor disease progression or improvement on patient sleep-disordered breathing. Pulmonologist evaluation is recommended to explore the need for respiratory support and airway clearance management if necessary. Also, perform a musculoskeletal evaluation for joint problems and scoliosis at least annually, implementing ankle-foot orthoses or assistant devices as needed.

Additionally, endocrinology evaluation is recommended to assess hypoglycemia every six months until two years [4]. Graham et al. have reported resolving transient neonatal hypoglycemia associated with hyperinsulinism resolved with diazoxide treatment [2]. Recommended multi-disciplinary plan of care is summarized in table 1.

**Table 1:** Recommended multi-disciplinary plan of care for Birk-Barel Syndrome The presented approach should be part of the multi-disciplinary evaluation in a sporadic genetic disorder with few identified cases in the world. Careful monitoring and evaluation of this entity are highly recommended for the early identification of associated comorbidities and the introduction of preventive measures to avoid complications. It is possible that some primary paediatricians may not be familiar with rare genetic disorders and may delay evaluation on an as-needed approach. More cases and further studies are needed to consider an as-needed approach to recommendations. EtCO_2_- End-tidal CO_2_, MFA- Mefanamic acid, FFA- flufenamic acid, NFA- niflumic acid, GERD- Gastroesophageal reflux disease,

Subspecialist	Considerations / Screening	Evaluation
Pulmonary	Screening for increased salivation, recurrent aspiration, atelectasis and gas exchange abnormalities.	Baseline radiographic imaging of the chest (CXR). Documentation of abnormal pulmonary sounds on examination. A record percentage of saturation. Revaluation bi-annually.
Sleep medicine	Screening for pediatric sleep disorders and nocturnal hypoxemia or hypoventilation.	Yearly diagnostic polysomnography with End-tidal CO2 (EtCO2). Flow or supplemental oxygen if needed. Actigraphy, sleep log and blood work to evaluate for gas exchange abnormalities. Consider treatment with flufenamic acid (FFA), niflumic acid (NFA) and Mefenamic acid (MFA).
Neurology	Evaluation for involuntary movement and seizures	An electroencephalogram should be considered if seizures are suspected.
Endocrinology	Baseline blood sugar and electrolytes levels in the setting of unexplained hypoglycemia and/or seizures.	Endocrinology referral to consider diazoxide therapy. Follow-up bi-annually up to age two.
Development	Early screening for global developmental delay focused on speech, physical and occupational therapy.	Additional referral including neurodevelopmental or neuropsychiatric evaluation may be considered.
Gastroenterology	Detection of Gastroesophageal reflux (GERD) disease and/or constipation.	Start GERD standard position precautions. Consider documentation of GERD severity with Upper GI and Barium Swallow or pH impedance studies.
Otorhinolaryngology	A baseline evaluation for upper airway obstruction.	Laryngoscopy evaluation to assess adenoid hypertrophy and consideration for adenotonsillectomy if indicated.
Nutrition	Monitoring of nutritional status and growth to ensure a BMI > 10^th^ percentile for age.	Caloric adjustment to meet nutritional goals. Recommendations about enteral feeding in patient with gastrostomy or nasogastric feedings.
Geneticist	Diagnostic testing for KCNK9 gene mutations and genetic counseling about family planning.	Referral to a genetic counselor for diagnostic discussion, prognostics and family planning.
Plastic Surgery	Assess for mandibular and palate abnormalities including retrognathia or cleft palate.	Cleft palate repair and/or mandibular distraction may be considered if indicated.
Ophthalmology	Evaluate for decrease lacrimation and corneal xerosis.	Annual evaluation. Lacrimal duct stent placement should be considered if indicated.
Orthopedics / Physical Medicine and Rehabilitation	Treatment as needed if scoliosis and/or extremity contractures are identified.	Annual spine series X-rays to monitor progression. Introduction of orthoses and assistive devices for early mobilization. Referral for occupation therapy.
Speech Pathologist	Evaluate for dysphagia, feeding difficulties and oro-sensory disorders. Use of special nipples if presence of palate abnormalities.	Modified Barium Swallow to rule out dysphagia and decrease aspiration risk.

Takahira et al. reported that three compounds of the nonsteroidal anti-inflammatory fenamic acid class of drugs: flufenamic acid (FFA), niflumic acid (NFA) and mefenamic acid (MFA), stimulate two-pore-domain potassium channels [9]. The reduced outward current through mutated TASK3 channels is partially rescued by FFA, suggesting that fenamic acid compounds have useful potential in treating this condition [2]. To date, two affected patients have been treated with oral MFA starting at age 14 months and were observed to have increased energy levels while on medication without documented adverse reactions [2]. However, clinical characteristics persisted despite MFA administration, and additional research is needed to predict the outcome and long-term effects of individuals treated.

Further studies may help to determine specific treatment guidelines with long-term sequelae documentation. Our patient demonstrated markedly improved levels of alertness, motor function, increased smiling frequency and interaction with caretakers after receiving respiratory support with NIV as a treatment for her severe sleep apnea. A combination of the aforementioned management recommendations with nonsteroidal anti-inflammatory fenamic acid drugs may provide increased patient benefits. However, the long-term effect of this combination is yet to be determined.

## Conclusions

KCNK9 imprinting syndrome is a sporadic disorder that requires a high index of clinical suspicion and genetic testing to confirm a diagnosis. Most cases reported to date, including our case, follow the same specific amino acid exchange. However, continued genetic testing is encouraged to elucidate possible alternative mechanisms of disease development. Treatment guidelines remain supportive, and further studies are needed to determine the long-term effects of proposed treatment combinations. Our patient exhibited marked improvement in her socio-motor function, levels of alertness, and smiling frequency after being placed in a regimen that also included respiratory support with NIV and an airway clearance regimen. A multi-disciplinary team approach is required to provide proper clinical care, improve quality of life and life expectancy.
